# Walking paths during collaborative carriages do not follow the simple rules observed in the locomotion of single walking subjects

**DOI:** 10.1038/s41598-022-19853-7

**Published:** 2022-09-16

**Authors:** Isabelle Maroger, Manon Silva, Hélène Pillet, Nicolas Turpin, Olivier Stasse, Bruno Watier

**Affiliations:** 1 LAAS-CNRS, UPS, CNRS, Université de Toulouse, Toulouse, France; 2 Institut de Biomécanique Humaine Georges Charpak, Arts et Métiers Sciences et Technologie, Paris, France; 3 Laboratoire IRISSE, EA4075, UFR des Sciences de l’Homme et de l’Environnement, Université de la Réunion, Le Tampon, France

**Keywords:** Data acquisition, Data processing, Computer modelling, Behavioural methods

## Abstract

Some works have already studied human trajectories during spontaneous locomotion. However, this topic has not been thoroughly studied in the context of human-human interactions, especially during collaborative carriage tasks. Thus, this manuscript aims to provide a broad analysis of the kinematics of two subjects carrying a table. In the present study, 20 pairs of subjects moved a table to 9 different goal positions distant of 2.7–5.4 m. This was performed with only one or both subjects knowing the target location. The analysis of the collected data demonstrated that there is no shared strategy implemented by all the pairs to move the table around. We observed a great variability in the pairs’ behaviours. Even the same pair can implement various strategies to move a table to the same goal position. Moreover, a model of the trajectories adopted by collaborating pairs was proposed and optimized with an inverse optimal control scheme. Even if it produced consistent results, due to the great variability which origins were not elucidated, it was not possible to accurately simulate the average trajectories nor the individual ones. Thus, the approach that was shown to be efficient to simulate single walking subjects failed to model the behaviour of collaborating pairs.

## Introduction

It is a great challenge to generate safe physical collaboration tasks between a robot and a human particularly with biped robots which are inherently unstable. To efficiently perform such collaborations, a good modeling of the human behaviour is necessary as it may turn the robot into a proactive partner which anticipates the human’s motions. However, providing to the robot an accurate model of its human partner is still regarded as a great challenge in Human–Robot Interaction (HRI)^1^. Thus, studying and modeling human motion is relevant in the HRI context. This study is part of the ANR-CoBot project which aims at achieving a table handling task in collaboration between a humanoid robot and a human. Thus, this paper focuses on the study of the behaviour of two humans carrying a table from a position to another in the objective of modeling their dynamics. The reliability of simulating their spontaneous trajectories in the aim of collaborating with a robot is also explored. Based on anterior works on the modeling of the spontaneous locomotion of single walking humans, the algorithm was adapted to simulate the pairs’ trajectories.

In biomechanics, collaborative carriage has already been studied from different angles.

On the one hand, some works focused on the walking gait of two individuals performing a collaborative task. For example, Fumery et al.^2^ studied the gait pattern of two subjects walking side by side while carrying a load. In this paper, the authors demonstrated that the Center of Mass (CoM) of each subject during a collaborative handling task follows the same pendulum-like behaviour than the one of a single subject walking without any load. In another work^3^, they also showed that the more a pair of subjects practised a carriage task, the more efficiently they performed it. Similarly, Lanini et al.^4^ studied the adjustment of the gait of two individuals interacting with one another. In contrast to the experiment proposed by Fumery et al., the authors instructed the two subjects to walk one in front of the other carrying a load. They concluded that, in most of the experiments, the subjects synchronized their walking gait during the carriage and that quadrupedal gait patterns, like pace, trot or diagonal sequence, emerged from the coupling of the two humans gait. A gait synchronization phenomenon was also observed by Sylos-Labini et al.^5^. In these experiments, the subjects, unable to see or hear each other, unintentionally synchronized their steps while they walked side-by-side with hand contact.

On the other hand, some works aimed to study the behaviour of the subjects during a collaborative task. Indeed, the forces shared through the haptic feedback can influence the subjects’ behaviour during the task. For example, in the context of collaborative target acquisition tasks, it was demonstrated that each member of the pair “specialized” in one specific role during the motion^6,7^. This result was obtained by looking at the recorded forces and motions. The leader handled the early part the motion when the follower managed the late part of the motion^8–10^. This is why during human–human haptic interaction experiments the two partners are commonly assigned a “leader” and a “follower” role^11,12^. Thus, when two partners interact through an object, they exchange haptic signals as internal force pattern which influence their behaviour during the collaboration. This is called haptic communication. Moreover, in Lanini et al.^12^, the focus was on basic human intention detection (start/stop and walk forward/backward) during a collaborative carriage. The authors recorded handling experiments where a deaf and blind subject, the follower, was connected to its partner, the leader, only through haptic feedback. Then, using this human–human interaction, a multiclass classifier was successfully trained to detect human intentions during load transportation. This demonstrated that the haptic communication allows the humans to infer their partner intentions which eases the conduct of the collaborative task.

However, to the best of our knowledge, none of the study about collaborative carriage focused on the spontaneous walking paths taken by the subjects. They have neither been analyzed nor modelled yet.

On the contrary, human walking paths out of collaborative tasks have already been studied in multiples works. Single walking human trajectories were recorded in various environments, with or without obstacles, to investigate the effect of the goal distance^13^, the foot placements^14^ or the relationship between the head and the trunk movements^15^ for example. Moreover, numerous works focused on the modeling and the simulation of single walking humans. Those studies went from the simulation of a 3D whole-body skeletal model with 42 degrees of freedom^16^ or more basic 2D or 3D models^17^ to the mere simulation of the CoM trajectory^18–20^. Human-like CoM trajectories can be generated using a non-holonomic system when a human is walking a straight line^18^. However, when he has to take sideways steps, a holonomic model is needed to better fit the human behaviour as it allows more degrees of freedom in the locomotion^13,21,22^. In Maroger et al.^22^, a holonomic model based on an Optimal Control (IOC) problem was designed to generate human-like trajectories between a given starting and goal positions. It was optimized using a bi-level Inverse Optimal Control (IOC) scheme, first introduced in Mombaur et al.^21^, to well fit average human CoM trajectories during locomotion. Using a similar OC model which takes as an input the recent past trajectory of a single walking human, it is even possible to accurately predict his future trajectory without knowing his goal position^23^. This kind of model, does not exist, as far as we know, for human walking during collaborative tasks.

Thus, the main goals of this work were threefold. First, there was a need to create a database of spontaneous human walking trajectories during carriage tasks. In this work, the tackled tasks were various table handling tasks. Those tasks were performed by pairs which were asked to act naturally. Experiments, detailed in the following section, were carried out to create such a database: 20 pairs performed a total of 1080 trajectories carrying a table all over the experimental room. Then, the recorded subject’s CoM trajectories, were analysed in order to better understand human behaviour during table handling tasks. This analysis investigated the emergence of a potential strategy which would be shared by all the pairs. In this paper, the word “strategy” names all the choices made by a pair when handling the table such as the path they choose to move the table to a given position or how they choose to place the table on a given position. A shared strategy emerges from all the experiments if most of the pairs make similar choices leading to similar behaviours. Finally, an OC based model was built to simultaneously generate the CoM trajectories of both subjects during table handling tasks. This model was designed using the same method as the human locomotion model described in Maroger et al.^22^. Thus, the model, introduced in this paper, was optimized to fit the average measured trajectories. The purposes of this approach are two-fold. First, this model aimed to be accurate and representative of most humans in order to, in future works, target a proactive HRI to carry a table. Then, it aimed to investigate the hypothesis that we could use the same method to simulate single walking humans and walking humans during a carriage task.

## Methods

### Experiments

#### Participants

Forty volunteers (15 females and 25 males) took part in these experiments with a mean (± standard deviation) age of $26.7 \pm 5.9$ years, weight of $71.7 \pm 14.6$ kg and height of $1.76 \pm 0.09$ m. They all were healthy subjects with no known pathological disorder likely to alter their gait. In order to preserve their natural behaviour, the participants were not briefed on the goals and expected results of this study. They were just informed about the experiments procedure and gave their written informed consent before taking part in this study. These experiments were conducted at the Centre de Ressources d’Expertise 2/21 et de Performance Sportive (CREPS) laboratory in Toulouse (France) in accordance with the declaration of Helsinki and with the approval of the University of Toulouse ethical committee.

The participants took part in the experiments as pairs. Once a subject assigned to a pair, the subject performed all the experiments with the same partner. Moreover, the twenty pairs were randomly formed without taking into account the physical features (weight and height) of the subjects.

#### Experimental protocol

**Figure 1:**
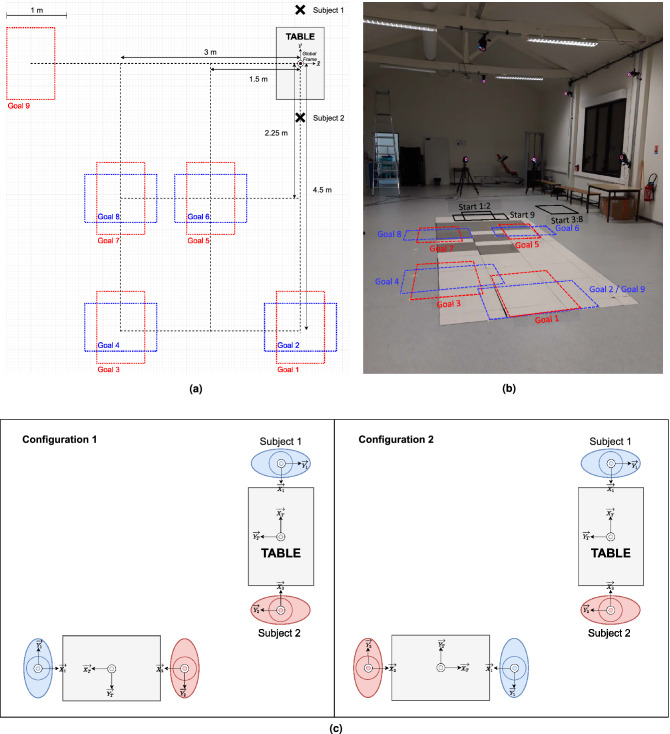
Experimental setup. (**a**) Representation of the 9 different goal positions. (**b**) Experiment room. The goal positions correspond to the ones represented in (**a**). (**c**) Two achievable configurations when handling the table to the goal positions 2, 4, 6 or 8. Each configuration is characterized by the orientation of the table on the given goal position.

The pairs were asked to carry a table to nine different goal positions and then return it to its starting position. Those positions are represented in Fig. 1a and their arrangement in the experiment room is shown in Fig. 1b. The goal positions were chosen in order to record table handling experiments including various destinations within a range of 2.7–5.4 m from the starting position and with different orientations. Furthermore, the table was the same for all the experiments. It measured $1.22 \times 0.8 \times 0.77$ m and weighted 20.7 kg. To limit non-haptic interactions between the subjects, they were asked not to talk to one another during the table transports.

During all the experiments, the subjects were instructed to walk at a self-selected casual pace. The experiment started with each member of the pair walking toward a table placed on a starting position. In every pair, one subject was appointed as *Subject 1* and the other as *Subject 2*. According to the label they were given, the participants were instructed to always grab the table on the same side. The starting positions of the two members of the pair according to their label are shown in Fig.1a. There was no given instruction on whether the subjects have to face the table or to stand their back to the table. Then, the pair grabbed and carried the table toward a goal position. In what follows this path are be named *forward path*. The goal positions were indicated on the floor with 4 pieces of adhesive tape, one for each table leg. The only instruction given to the participants was that all the table legs must match with the markers on the floor, there was no instruction on the orientation of the table at the end of the handling. So for each goal position, there were two possible configurations for the table. The configuration was characterized by the orientation of the table on the given goal position. An example is illustrated in Fig.1c. Once the table has reached the goal position, the subjects laid the table on the floor and released it. Then, the pair grabbed the table again and carried it back to the starting position. This path are be named *return path*. Once the table has reached the starting position, the subjects laid the table on the floor and released it. The trial was over.

The nine table handling experiments were performed 3 times by each pair following three different scenarios:

- Scenario 1: Only Subject 1 knows the goal position.
- Scenario 2: Only Subject 2 knows the goal position.
- Scenario 3: Both subjects know the goal position.

In the following, the subject which knows the goal position is called the *leader* while the other one is called the *follower*, in accordance with the literature. In the third scenario, both subjects are called *leaders*. Thus, in this manuscript, *leader* refers to the knowledge of the target rather than the rule of one subject over the other one. Within a scenario, the goal positions were randomly given to the leader in order to avoid the follower to anticipate where the pair has to bring the table. For the same reason, the participants were instructed not to communicate during the table handling.

However, the three scenarios were not carried out randomly, they were performed one after the other. Moreover, when the pair returned the table to its starting position, both subjects knew where they have to carry the table. This is why, in those cases, the scenario was always the third one whatever the scenario dictated to go to the goal position was. Thus, each pair performed a total of 54 trajectories (9 goals $\times$ 2 paths $\times$ 3 scenarios). In the end, 1080 trials were performed by the 20 pairs involved in this experiment. This results in 3240 measured trajectories of subjects and of the table.

#### Data collection

3D kinematic data of the subjects and of the table were recorded using a motion capture system (15 infrared VICON cameras sampled at 200 Hz). Each subject wore 14 passive markers. 4 markers were placed on their pelvis, two on the postero-superior iliac spines and two on the antero-superior iliac spines. 6 other markers were put on the feet of the subjects in order to record their footsteps and the last four markers were put on their head. Regarding the table, its CoM positions and orientations were recorded using three passive markers placed on three of the four corners of the table. The experiment room is shown in Fig. 1b.

### Analysis

#### Data processing

The kinematic data were filtered using a fourth order, zero phase-shift, low-pass Butterworth with a 10 Hz cutoff frequency. Then, the recorded data from all the experiments were sequenced in three sections: walk to the table, table handling during the forward path and table handling during the return path. This sequencing was based on the height of the table. The onset and offset of each motion were determined as when the height of the table exceeded and returned below 5 mm from the floor, respectively. Finally, the kinematic data of interest, which are, in this manuscript, the CoM positions and orientations of the two subjects and of the table, were computed for every extracted sequence. The horizontal position (*x*, *y*) of the CoM of the subjects and the orientation $\theta$ of their pelvis, with respect to the global frame were computed using the four markers placed on the pelvis of the subjects according to a previously published methodology^24^. For one experiment, those datasets are called *the measured trajectories* and denoted $X_1$, $X_2$ and $X_T$ for Subject 1, Subject 2 and the table respectively. In what follows, the measured trajectories for the *j*th pair ($j \in \llbracket 1,20 \rrbracket$) during the *k*th scenario ($k \in \llbracket 1,3 \rrbracket$) is denoted $$X^{mes,k}_j = \begin{pmatrix}X^{mes,k}_{j,1}&...&X^{mes,k}_{j,N} \end{pmatrix}$$ with $X \in \{X_1,X_2,X_T\}$, with *N* the number of measurements in this trajectory and $X^{mes,k}_{j,i} =(x^{mes,k}_{j,i},y^{mes,k}_{j,i},\theta ^{mes,k}_{j,i}), \, \forall i \in \llbracket 1,N \rrbracket$.

The CoM trajectories were analyzed after normalizing the time from 0 to 100% on 500 points. Thus, in this paper $N = 500$. Using the normalized trajectories, the *average trajectories* (arithmetic mean) were computed for the two subjects and the table for every goal position with the two different configurations and for every scenario. They are denoted $$\bar{X}^{mes,k} = \begin{pmatrix}\bar{X}^{mes,k}_{1}&...&\bar{X}^{mes,k}_{N} \end{pmatrix}$$ with $\bar{X}^{mes,k}_i = \frac{1}{20} \sum _{j=1}^{20} X^{mes,k}_{j,i}, \quad \, \forall i \in \llbracket 1,N \rrbracket$.

#### Data analysis

The goal of this data analysis was to study the strategies implemented by the pairs to move the table. In this context, the analysis focused on the trajectories and the configurations chosen by the pairs for all the experiments. First, we wanted to figure out if the choices made by one pair to handle the table depend on the given scenario or on the direction of the motion (forward or return path). Then, this study focused on the optimality of the strategy chosen by the pairs. The final aim of this analysis was to determine whether or not a shared strategy emerges from all the experiments, i.e. whether or not the majority of the pair makes similar choices while carrying a table. For such analysis, we chose to compare the average trajectories with the variety of the measured trajectories. Indeed, we made the assumption that, if the average trajectories are representative of all the performed trajectories, it can be stated that all the pairs have a non-distinguishable behaviour and that a shared optimal strategy globally emerged spontaneously as already observed for individuals^22^. Thus, this data analysis tackled four main topics and aimed to answer the following questions:

- Differences between scenarios: Is there a scenario where the pair moves the table faster than in the others? (Q1.1) Does a pair choose a similar path to carry the table to the same goal position during different scenarios? (Q1.2) Does the path taken by a pair when there is two leaders closer to the path chosen when Subject 1 was the leader or when Subject 2 was the leader? (Q1.3)
- Differences between the forward and the return paths: Are the forward paths similar to their respective return paths? Are the trajectories during carriage task asymmetrical like those of a single walking human^21^? (Q2.1) Is one path performed faster than the other? (Q2.2)
- Optimality of the chosen configuration: Does a subject tend to choose the configuration that allows him to travel the minimal distance? That allows their partner to travel the minimal distance? Or that allows the pair as a whole to travel the minimal distance? (Q3)
- Variability of the trajectories: Is there a great variability between pairs? (Q4.1) or even within a pair? (Q4.3) Are the average trajectories representative of all the pairs for all the trajectories? (Q4.2) Does the distance to the goal increase the variability of the performed trajectories? (Q4.4)

To answer those questions, some parameters needed to be introduced:

- Travel time was defined as the elapsed time between the moment the table takes off the floor and the moment it lies on the floor again.
- Travelled distance was defined as the euclidean distance between the starting position $(x_{i,s},y_{i,s})$ and the goal position $(x_{i,f},y_{i,f})$ of Subject 1 ($i=1$) or Subject 2 ($i=2$) or the table ($i=T$) :
  1 $$\begin{aligned} D_i = \sqrt{(x_{i,f}-x_{i,s})^2+(y_{i,f}-y_{i,s})^2} \end{aligned}$$
  One can denote that this distance is not the exact distance travelled by the subjects as they never chose the straight path. The travelled distance of the table $D_T$ is named *global distance* in what follows.
- Distance between curves: To determine if a chosen path was “similar” to another, a metrics needed to be defined. This metrics needed to assess if the geometric trajectories were close from one another and if the orientations of the subjects were alike. This is why, using the metrics introduced in Maroger et al.^22^ was suitable. Given 2 trajectories $X_1$ and $X_2$, the following *linear* and *angular distances*, respectively $d_{xy}$ and $d_\theta$, were computed as follows:
  2 $$\begin{aligned} \left\{ \begin{array}{l} d_{xy}(X_1,X_2) = \frac{1}{N} \sum _{i=1}^N \sqrt{(x_{1,i}-x_{2,i})^2 + (y_{1,i}-y_{2,i})^2}\\ d_{\theta }(X_1,X_2) = \frac{1}{N} \sum _{i=1}^{N} |\theta _{1,i}-\theta _{2,i} |\end{array} \right. \end{aligned}$$
  With those definitions, the smaller the distances are, the closer the compared trajectories are. The linear and angular distances between the paths taken by the same pair during different scenarios, between the forward and return paths and also between the average and the measured trajectories were computed.
- Symmetry between the forward and the return path: In this manuscript, the smaller the linear and angular distances between the forward path and its respective reversed return path were, the more symmetrical a forward path and a return path might be considered.
- Optimal configuration: The chosen configuration was identified by measuring the orientation of the table on its goal position. The used criteria to tell if the chosen configuration was optimal with respect to another was based on the travelled distance by each of the subjects and by the whole pair. Thus, the chosen configuration was qualified as optimal for Subject 1, Subject 2 or the pair if it respectively minimized the distance travelled by Subject 1 ($D_1$), by Subject 2 ($D_2$) or the sum of the distance travelled by the two subjects ($D_1 + D_2$). Let us denote that, for Goal 2 (see Fig. 1a), both subjects walked the same distance regardless of the configuration they chose. In that specific case, the two configurations were regarded as optimal.

### Modeling

Another purpose of this paper was to model the walking trajectories of two humans carrying a table. Accurately model the subjects’ behaviour is a first step toward the prediction of their motion during a table handling task. To achieve this goal, an OC model optimized through an IOC scheme to fit the average measured trajectories was introduced. As a reminder, the choice to model average trajectories instead of individual ones came from the hypothesis that the same method could be used to accurately simulate single walking humans and walking humans carrying a table. In this section, the focus is on the modeling of the subjects’ average trajectories during Scenario 3 only. We did not optimize the model for the other scenarios. We chose to only focus on Scenario 3 because both forward and return paths were performed in this scenario and, thus, it multiplied the number of trajectories to fit for the inverse optimization.

#### Model definition

The OC model introduced in Maroger et al.^22^ was adapted to simultaneously generate the CoM trajectories of the two subjects. Originally this model was designed to generate the trajectories of one human walking alone without constraints. Here, the model was modified in order to simulate the trajectories of two humans coupled through a table handling task. This new model is named *coupled OC model*. Thus, the dynamics of the system is not the dynamics of one holonomic system anymore, but the dynamics of two holonomic systems:

3 $$\begin{aligned} \left\{ \begin{array}{l} \dot{x_1} = \cos {\theta _1} \times v_{1,forw} - \sin {\theta _1} \times v_{1,orth} \\ \dot{y_1} = \sin {\theta _1} \times v_{1,forw} + \cos {\theta _1} \times v_{1,orth} \\ \dot{\theta _1} = \omega _1 \\ \dot{v}_{1,forw} = u_{1,1} \\ \dot{v}_{1,orth} = u_{1,2} \\ \dot{\omega _1} = u_{1,3} \end{array} \right. \left\{ \begin{array}{l} \dot{x_2} = \cos {\theta _2} \times v_{2,forw} - \sin {\theta _2} \times v_{2,orth} \\ \dot{y_2} = \sin {\theta _2} \times v_{2,forw} + \cos {\theta _2} \times v_{2,orth} \\ \dot{\theta _2} = \omega _2 \\ \dot{v}_{2,forw} = u_{2,1} \\ \dot{v}_{2,orth} = u_{2,2} \\ \dot{\omega _2} = u_{2,3} \end{array} \right. \end{aligned}$$

**Figure 2:**
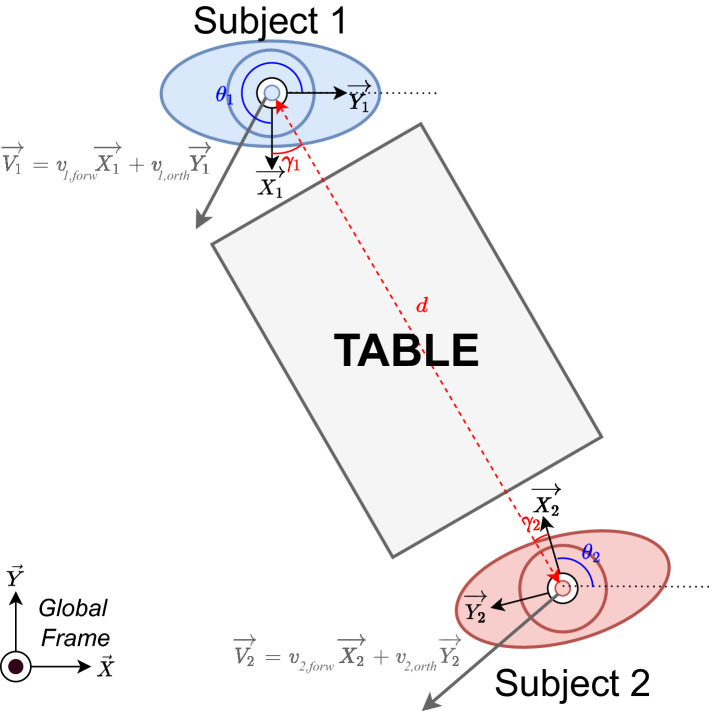
Coordinate systems and orientations in the trajectory problem to solve.

$(x_i,y_i)$ is the generated position of the CoM in the horizontal plane of the subject *i* with $i \in \{1,2\}$. $\theta _i$ is the generated orientation of its pelvis in the global frame. $v_{i,forw}$ and $v_{i,orth}$ are the tangent and orthogonal velocities with respect to the orientation of its pelvis and $\omega _i$ is its angular velocity. All those variables are represented in Fig. 2. The state of the coupled OC model is $X = (X_1,X_2)^T = ((x_1,y_1,\theta _1),(x_2,y_2,\theta _2))^T$ and the control is $U = (u_{1,1},u_{1,2},u_{1,3},u_{2,1},u_{2,2},u_{2,3})^T$. So, this new model counts twice as many variables as the OC model presented in Maroger et al.^22^. The problem is of the following form and was solved using a Differential Dynamic Programming (DDP) solver^25^ from the Crocoddyl library^26^ :

4 $$\begin{aligned} \min _{X(.),U(.),T} \int _0^T \phi _r(X(t),U(t)) \, \mathrm {d} t + \phi _t(X(T)) \end{aligned}$$

with $\phi _r$ and $\phi _t$ the running and terminal cost functions. *T* is the time needed to go from the starting position to the goal position. This problem was solved under the following constraints:

5 $$\begin{aligned} \left\{ \begin{array}{l} \dot{X}(t) = f(X(t),U(t)) \text {Dynamical constraint} {\rm (Eq. {3})} \\ X(0) = X_s \text {Initial constraint} \end{array} \right. \end{aligned}$$

with $X_s=(x_{1,s},y_{1,s},\theta _{1,s},x_{2,s},y_{2,s},\theta _{2,s})^T$ the starting state.

The cost functions are as follows:

6 $$\begin{aligned} \left\{ \begin{array}{l} \phi _r(X(t),U(t)) = \alpha _0+\alpha _1 u_{1,1}^2(t)+\alpha _2 u_{1,2}^2(t)+\alpha _3 u_{1,3}^2(t) + \alpha _4 u_{2,1}^2(t)+\alpha _5 u_{2,2}^2(t)+\alpha _6 u_{2,3}^2(t) +\alpha _7 \psi _1(X(t),X_f)^2\\ + \alpha _8 \psi _2(X(t),X_f)^2 +\alpha _9 \chi (X(t))+ \alpha _{10} (\xi _1(X(t))+\xi _2(X(t)))\\ \phi _t(X(T),U(T)) = \beta _0 ((x_{1,f}-x_1(T))^2+(y_{1,f}-y_1(T))^2+(x_{2,f}-x_2(T))^2+(y_{2,f}-y_2(T))^2) +\beta _1 ((\theta _{1,f}-\theta _1(T))^2 \\ +(\theta _{2,f}-\theta _2(T))^2) + \beta _2(v_{1,forw}(T)^2+v_{1,orth}(T)^2 +v_{2,forw}(T)^2+v_{2,orth}(T)^2) + \beta _3 (\omega _1(T)^2+\omega _2(T)^2) \end{array} \right. \end{aligned}$$

In this equation, $\psi _i(X(t),X_f) = \arctan \frac{y_{i,f}-y_i(t)}{x_{i,f}-x_i(t)}-\theta _i(t)$ with $i \in \{1,2\}$ and $X_f = (x_{1,f},y_{1,f},\theta _{1,f},x_{2,f},y_{2,f},\theta _{2,f})$ the final state. Then, $\chi$ and $\xi _i$ are exponential barrier functions. This means that they enforce some physical constraints of the system in the cost function. Indeed, the $\chi$ function imposes that the subjects are separated with a distance between $1.6 \,$ and $2.1 \, \mathrm{m}$ due to the table length and the $\xi _i$ function imposes that the difference between the orientation of the vector from the subject *i* to the subject *j* and the orientation of the subject *i* does not exceed $\frac{\pi }{3}$ rad. In other words, those constraints respectively mean that both subjects hold one side of the table and that the subjects almost face each other. The coupling of the two subjects is implemented through these exponential barriers. Mathematically, they can be written as follows : $$\chi (X(t)) = \left\{ \begin{array}{l} 0\text { if }1.6< d = \sqrt{(x_1(t)-x_2(t))^2 + (y_1(t)-y_2(t))^2} < 2.1\\ \exp {(\min {(|d-2.1|,|d-1.6|)})} -1\text { otherwise } \end{array} \right.$$ and $$\xi _i(X(t)) = \left\{ \begin{array}{l} 0\text { if } \gamma _i = | \arctan \frac{y_j - y_i}{x_j-x_i} - \theta _i | < \frac{\pi }{3}\\ \exp {(\gamma _i-\frac{\pi }{3})} -1\text { otherwise } \end{array} \right.$$ with $j \in \{1,2\}$ and $j \ne i$. *d* and $\gamma _i$ are shown in Fig. 2. Moreover, $\alpha = (\alpha _0,\alpha _1,\alpha _2,\alpha _3,\alpha _4,\alpha _5,\alpha _6,\alpha _7,\alpha _8,\alpha _9,\alpha _{10})$ are the weights of the running cost and $\beta = (\beta _0,\beta _1,\beta _2,\beta _3)$ the weights of the terminal cost. Both $\alpha$ and $\beta$ were first determined with the IOC scheme described below.

In what follows, the trajectories generated with this coupled OC model for Subject 1 and Subject 2 are respectively denoted $X^{gen}_1$ and $X^{gen}_2$.

#### IOC

To allow this model to fit well the average human measurements, it was needed to optimize the cost function weights $\alpha$ and $\beta$ with an IOC scheme as in Maroger et al.^22^. This scheme was first introduced in Mombaur et al.^27^ The purpose of this method was to find the weights of the cost function, described in Eq. (6), which allowed the OC model to generate trajectories that best fit the mean measured trajectories. This bi-level method finds the optimal weights of the OC problem solving another optimization problem defined as follows:

7 $$\begin{aligned}&\min _{\alpha ,\beta } \frac{1}{N_{traj}} \sum _{n=1}^{N_{traj}} d_{xy_{1,n}}(\bar{X}^{mes,3}_{1,n},X^{gen}_{1,n}(\alpha ,\beta )) + d_{xy_{2,n}}(\bar{X}^{mes,3}_{2,n},X^{gen}_{2,n}(\alpha ,\beta )) \nonumber \\&\quad + \frac{1}{2} ( d_{{\theta }_{1,n}}(\bar{X}^{mes,3}_{1,n},X^{gen}_{1,n}(\alpha ,\beta )) + d_{{\theta }_{2,n}}(\bar{X}^{mes,3}_{2,n},X^{gen}_{2,n}(\alpha ,\beta )) ) \end{aligned}$$

with $N_{traj}$ the number of measured trajectories that the model tries to fit. Here $N_{traj} = 31$ as the IOC scheme was performed using all the average forward paths and average return paths measured during Scenario 3. Let us denote that there was more than 9 paths for each direction (13 forward paths and the 18 return paths) because the table was not always placed in the same configuration for every trial. Furthermore, we hypothesized that for the return paths Subject 1 became Subject 2 and reversely. Indeed, broadly speaking, during the experiments, Subject 1 faced the goal position while Subject 2 faced the starting position (see Fig. 1a). So, when performing the forward path, Subject 1 faced the position where the table had to be placed and, conversely, when performing the return path, Subject 2 faced this position. Thus, we made the assumption that the behaviour of the subjects were switched between the forward and the return path. Otherwise, let us denote that the cost function in Eq. (7) was a weighted sum in order to make the linear and the angular distance of the same magnitude.

The IOC problem was solved with a derivative free method called the Powell method^28^ provided by the Scipy library^29^. More details about this IOC scheme can be found in Maroger et al.^22^.

Once the optimal weights found, the linear and angular distances between the generated and the measured trajectories were computed in order to assess the ability of the OC model to fit the average measured data.

### Statistical analysis

All the statistical tests described below were performed using the Python Scipy library^29^.

To compare the scenarios, the forward and return paths, the pairs or the measurements and the generated trajectories, distances between curves, as defined in the Data analysis section, were computed. As the data were not normal according to a Shapiro–Wilk test, only non-parametric tests were used. First, Kruskal–Wallis tests were used to assess the differences between:

- The travel time performed during the different scenarios.
- The linear and angular distances between the forward and return path for the subjects and for the table.
- The linear and angular distances between the average trajectories and the measured forward and return paths during Scenario 3.
- The linear distances between the average trajectories and the measured trajectories for all the pairs.
- The linear distances between the different return paths that a pair performed to go back from the same goal.
- The linear and angular distance between the average trajectories and the measured trajectories during Scenario 3 and the linear and angular distance between the average trajectories and the trajectories generated with the coupled OC model.

Then, Mann–Whitney U rank tests were performed for a side-by-side comparison of the data. Significance of all those tests was set at $p < 0.05$.

Futhermore, Fisher’s exact test were used to determine if the chosen trajectories during Scenario 3 and the linear distances between the different return paths that a pair performed to go back from the same goal depend on the subject. The same test was also used to check that the choices of configuration between the different scenarios were not random.

Moreover, some of those distances are represented in form of box and whisker plots in what follows. Those plots show the median, the lower and upper quartile values ($Q_{low}$ and $Q_{up}$) and extend from the maximum to the minimum within $[Q_{low}-1.5(Q_{up}-Q_{low}),Q_{up}+1.5(Q_{up}-Q_{low})]$. The other printed values are considered as outliers.

## Results

**Figure 3:**
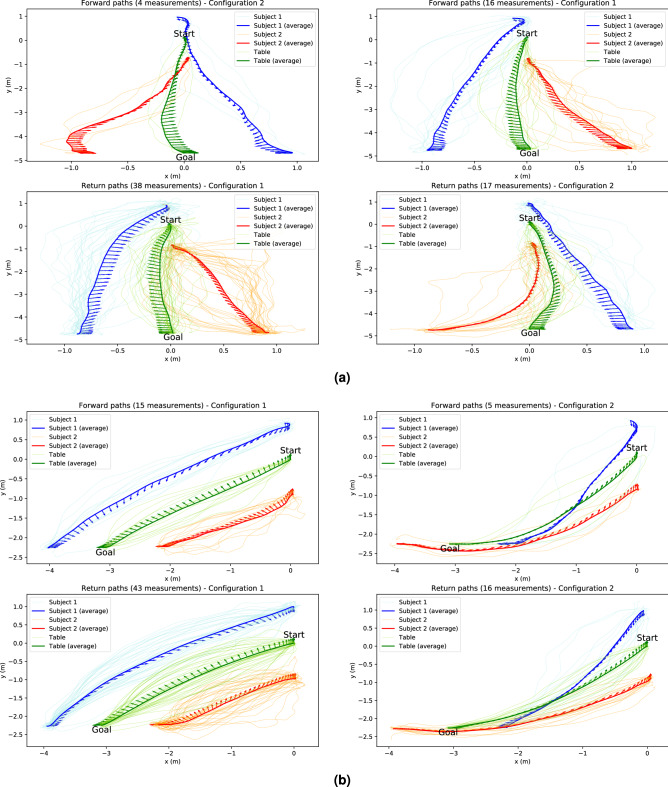
Trajectories performed by each pair and average trajectories during Scenario 3 for two different goals. The trajectories performed for these two goals are typical of the observed straight (Goal 2) and oblique (Goal 6) motions. The arrows represent the average orientation of the pelvis of Subject 1 (in blue) and Subject 2 (in red) and of the table (in green) during locomotion. (**a**) Trajectories toward Goal 2. (**b**) Trajectories toward Goal 6.

Examples of average trajectories for the forward and return paths toward two different goals for Scenario 3 are represented in Fig.3a,b.

### Differences between scenarios

**Figure 4:**
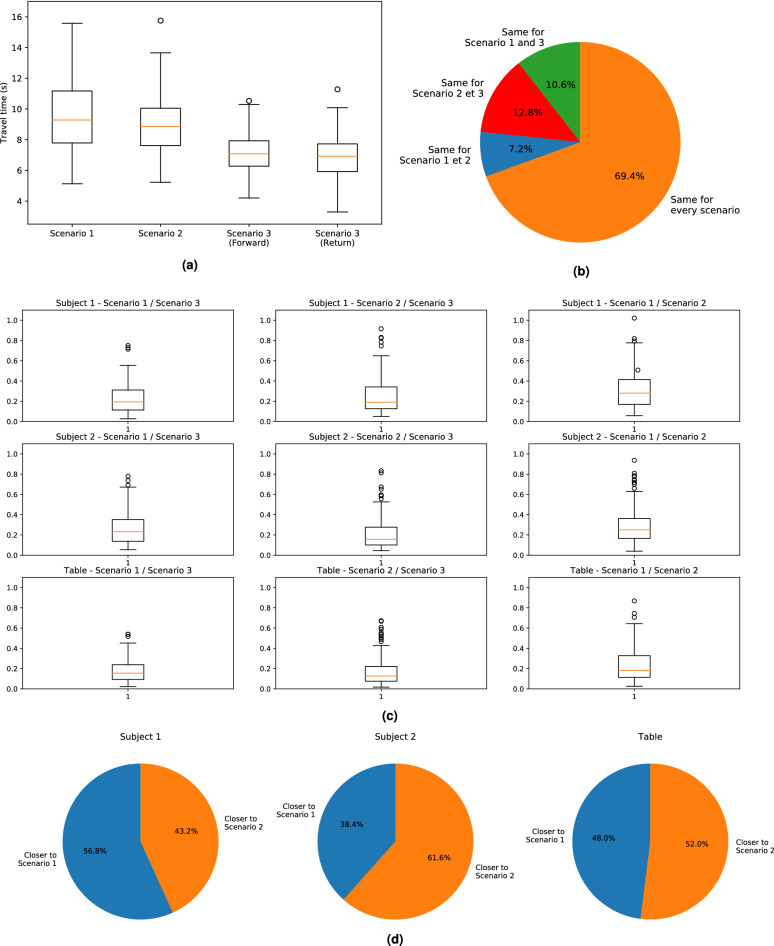
Difference between different scenarios for all pairs. (**a**) Boxplot of the travel times according to the different scenarios. For Scenario 3, the boxplots for the forward and return paths were split in order to determine if the pairs move faster or slower during the forward path or the return path. (**b**) At the end of each forward path, every pair laid the table in the configuration they chose. This pie chart shows the percentage of experiments where the pairs put the table in the same configuration during Scenario 1 and 3 but not during 2 (green), where they put it in the same configuration during Scenario 2 and 3 but not during 1 (red), where they put it in the same configuration during Scenario 1 and 2 but not during 3 (blue) and where they always put it in the same configuration during the three scenarios (orange). (**c**) Boxplots of the linear distances (m) between the trajectories performed during Scenario 1 and 3 (on the left), Scenario 2 and 3 (in the middle) and Scenario 1 and 2 (on the right) for Subject 1, Subject 2 and the table. (**d**) Closeness of the trajectory performed in the Scenario 3 with the two others according to the linear distance for Subject 1, Subject 2 and the table.

(Q1.1) The travel time of all the forward and return paths are represented in Fig.4a. There is a significant difference between the scenarios where only one subject knows the goal position and the scenario where both partners know it (Kruskal–Wallis test, $p < 0.001$). Indeed, the pairs performed the carriage task faster when each member of the pair knows the goal, namely in the third scenario . The travel time in the first and second scenario are not similar (Mann–Whitney test, $p < 0.05$). The experiments with the Subject 2 as a leader were performed sightly faster than the ones with the Subject 1 leader with an average travel time of $8.9 \pm 1.8 \, \mathrm{s}$ versus $9.6 \pm 2.2 \, \mathrm{s}$.

(Q1.2) Then, to study the impact of the different leaderships on the table configuration, the chosen configurations were analysed for each scenario. The choice of configuration is represented in Fig. 4b. This plot shows that, for the major part of the experiments (about 70%), when placing the table on a same goal position the pairs chose the same configuration. When it was not the case, the choice does not seem to depend on the scenario. Thus, the chosen configuration did not rely on the leader. Moreover, the analysis of the chosen configurations for each pair supports this results. Indeed, while 10% of the pairs always placed the table in the same configuration for the three scenarios, the others often changed the configuration for the carriage to the same goal during different scenarios. Moreover, for every goal positions, only one pair never placed the table in one same configuration for the three scenarios.

(Q1.3) For a better understanding of the differences of the subjects’ behaviour according to every scenario, we computed the linear distance between the trajectories taken during the different scenarios for all the pairs. Naturally, only the trajectories leading to the same final configuration of the table were compared. Thus, for every pair and every forward path to the nine different goal positions, the linear distances between the trajectories performed in the different scenarios by Subject 1, Subject 2 and the table were computed and are represented in Fig. 4c. These results demonstrated that all the scenarios were equally distant from each other. In other words, similar differences are observed between the paths performed during the different scenarios. However, when focusing on the Scenario 3, we can highlight that the paths of the Subject 1 were significantly closer to the one spontaneously chosen in the Scenario 1 whereas the paths of the Subject 2 were closer to the path spontaneously chosen in the Scenario 2 (Fisher test, $p < 0.05$). The paths of the table in scenario 3 presented similar closeness to Scenarios 1 and 2 (Fig. 4d). To conclude, when both partners knew the goal they tended to follow the path they spontaneously chose when they were alone to know the goal. Thus, the position of the subjects with respect to the table seems to bias the chosen trajectories when both partners are leaders. In spite of the significance of the observed differences, this strategy was true only for $60\%$ of the trials and thus cannot be generalized. Indeed, for some pairs, during the Scenario 3, the subjects were closer to the paths they took when they were not the leaders. Obviously, this result alleviated the previous conclusion.

### Differences between the forward and the return paths

**Figure 5:**
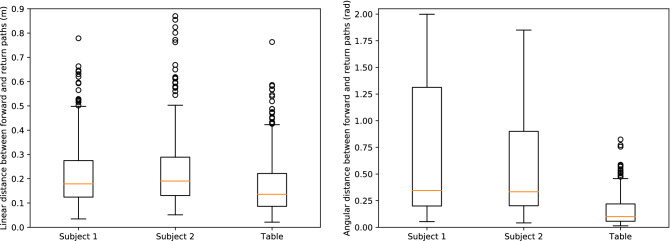
Boxplots of the linear (on the left) and angular (on the right) distances between forward and return paths for Subject 1, Subject 2 and the table trajectories for all pairs.

(Q2.1) The boxplots of the linear and angular distance between the forward and theirs respective return paths for Subject 1, Subject 2 and the table are represented in Fig. 5. The linear distances exhibited means of $0.21 \pm 0.12$ m, $0.23 \pm 0.15$ m and $0.17 \pm 0.12$ m for Subject 1, Subject 2 and the table trajectories. While the means of the angular distances respectively amounted to $0.69 \pm 0.59$ rad, $0.56 \pm 0.47$ rad and $0.15 \pm 0.13$ rad. Those data suggested that most of the forward and return paths performed by Subject 1 and Subject 2 were asymmetrical while the paths taken by the table might be close to be symmetrical in most of the cases. The linear and angular distances for Subject 1 and 2 are significantly different from the one for the table (Mann-Whitney test, $p < 0.001$).

(Q2.2) Otherwise, the travel time during the forward paths during Scenario 3 and all the return paths were similar (Mann–Whitney test, $p > 0.1$) as represented in Fig.4a. So, when both partners knew where the goal position was, they performed the carriage task as swiftly during the forward path as during the return path.

### Optimality of the chosen configuration

**Figure 6:**
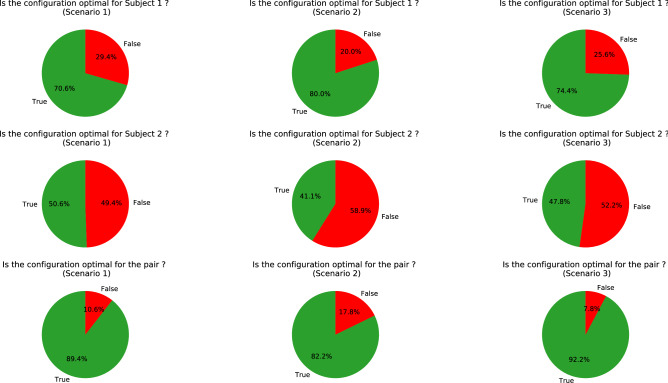
When the table configuration minimizes the travelled distance of Subject 1, Subject 2 and the pair, it is called optimal. These pie charts show the percentage of experiments where the pair put the table in an optimal configuration for Subject 1 (at the top), for Subject 2 (in the middle) and for the pair (at the bottom) according to the different scenarios.

(Q3) For every forward path, the travelled distance of the two subjects were computed and labelled as optimal or not optimal for each subject and for the whole pair. The results of this study are shown in Fig. 6. First, the choice of configurations during the different scenarios cannot be considered random (Fisher test, $p> 0.05$ for Subject 1, Subject 2 and the pair). Then, similar results were acquired in every scenario. Indeed, in every scenario, Subject 1 mostly walked the shortest possible distance while Subject 2 took the longest path in almost half of the experiment even more when the subject is the leader. This means that the position of the subjects with respect to the table position affected more the path they took than being or not the leader of the pair. Moreover, it can be denoted that the pair chose the optimal configuration for the whole pair in most of the cases. To conclude, the pair seemed to mostly choose the optimal path for the whole pair at the expense of the very same subject. Their collective strategy favored the group rather than the individuals.

**Figure 7:**
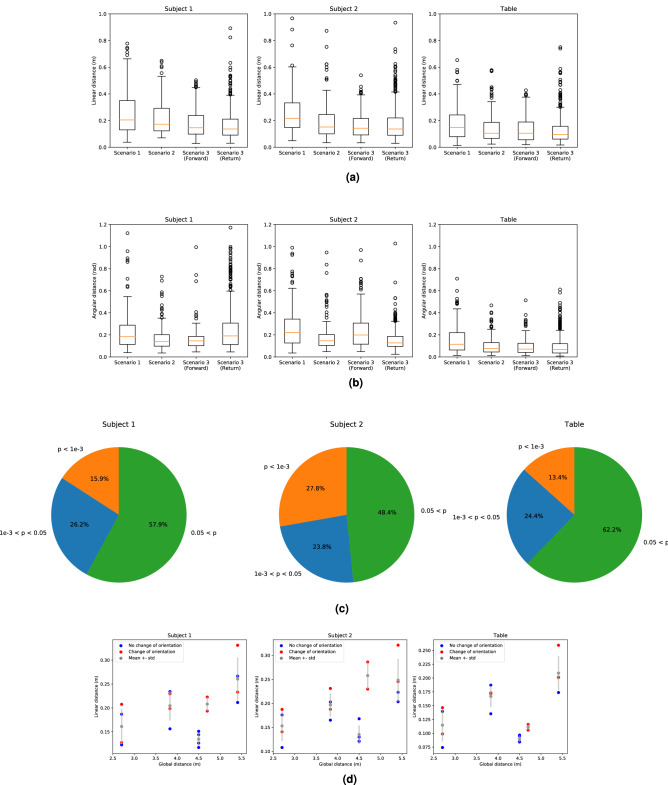
Variability of the performed trajectories. (**a**) Boxplot of the linear distances between the average trajectories and the measured trajectory for all goal positions according to the different scenarios. (**b**) Boxplot of the angular distances between the average trajectories and the measured trajectory for all goal positions according to the different scenarios. (**c**) The pairs returned the table 3 times in the same conditions from each goal position. Thus, we can compute the linear distances between these 3 returns for every pair and every goal. Kruskal–Wallis tests were performed to assess if these distance are significantly different. These pie charts show the results of these statistical tests. (**d**) Mean linear distances between the average trajectories and the measured trajectories according to the global distance . In blue are plotted the distances for the trajectories which require a change of the table’s orientation (Goal 2, 4, 6, 8) while the others are plotted in red. Let us denote, that there is an even number of points of each color for every global distances because there are two achievable configurations per goals.

**Figure 8:**
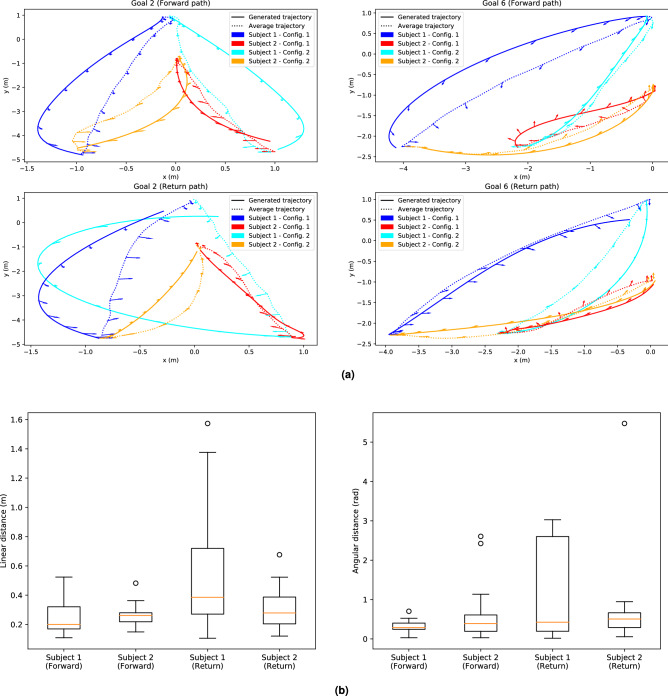
Comparison between the trajectories generated with the coupled OC model and the average measured trajectories. (**a**) Average (dotted line) and generated trajectories (full line) for both subjects, with both configurations when measured. The arrows represent the orientation of the pelvis of the subjects during locomotion. (**b**) Boxplot of the linear (on the left) and angular (on the right) distances between the generated and the average measured trajectories for every goal positions according to both subjects and to the direction of the motion.

### Variability of the trajectories

(Q4.1) First of all, the pair trajectories represented in Fig. 3a,b seem to show a great range of behaviours. The mean of the linear distances between the average trajectory and the measurements for Subject 1, Subject 2 and the table respectively amounted to $0.20 \pm 0.14$ m, $0.20 \pm 0.14$ m and $0.15 \pm 0.12$ m, while the mean angular distances were equal to $0.20 \pm 0.16$ rad, $0.21 \pm 0.15$ rad and $0.11 \pm 0.10$ rad. Moreover, the maximum linear distances for Subject 1, Subject 2 and the table respectively were 1.28 m, 1.27 m and 1.32 m and the maximum angular distances are 1.12 rad, 0.99 rad and 0.71 rad. The boxplots of those distances according to the different scenarios are represented in Fig.7a,b. Furthermore, the boxplots in the Scenario 3 for the forward and the return path looked alike. This is corroborated by a statistical test which revealed no significant differences between the linear distances (Kruskal–Wallis test, $p > 0.1$ for Subject 1, Subject 2 and the table).

(Q4.2) Then, we computed the linear distance between the average and the measured trajectories according to the pairs. Most of the pairs exhibited a boxplot similar to the one shown in Fig.7a while other present significant differences with greater medians and quartile values (Kruskal–Wallis test, $p < 0.001$ for Subject 1, Subject 2 and the table). Thus, the pairs demonstrated various behaviours.

(Q4.3) It is important to notice that a great variability also existed among the trajectories performed by a same pair. Indeed, the same return path was measured three times for each goal positions and for each pair and we performed a Kruskal–Wallis test to assess their differences. The pie chart represented in Fig.7c shows the p-value computed during those Kruskal-Wallis tests. They show that around 42%, 52% and 38% of the return paths of respectively Subject 1, Subject 2 and the table have a p-value lower than 0.05. This means that around half of the returns from the same goal position performed by a same pair were significantly different. Moreover, it is interesting to denote that those percentages did not depend on whether we look at the trajectories performed by Subject 1, Subject 2 or the table (Fisher test, $p > 0.1$). So when a pair demonstrated such variability in its own behaviour, it is not surprising that the variability between pairs was even bigger.

(Q4.4) Finally, the linear distances between the average and the measured trajectories is plotted with respect to the global distance to be covered in Fig. 7d. Another information is held on this plot: the markers are of different color whether the table orientation is the same or not at the beginning and at the end of the motion. For example, the orientation of the table changed for all the experiment performed with the goal position represented in blue on Fig.1a. This plot seems to show that the mean linear distance between the average and the measured trajectories increased when the global distance increased and when the table was moved along both $\vec {x}$ and $\vec {y}$ axis. Indeed, when the global distance was equal to 4.5 and 4.7 m, the goal positions were Goal 1,2 or 9 which only matched motion along one axis. In those cases, the increase according to the global distance did not emerge. Moreover, at a constant global distance, the linear distance was a little bit higher in average when the table change of orientation between the start and the end of the motion. However, there was not enough data to state that those results are always true.

### Model assessment

#### IOC results

Once the coupled OC problem introduced, the weights of the cost functions introduced in Eq. (6) were optimized using the IOC problem defined in Eq. (7). Thus, the optimal weights which allowed the generated trajectories to best fit the average trajectories for Scenario 3 are:

8 $$\begin{aligned} \left\{ \begin{array}{l} (\alpha _0,\alpha _1,\alpha _2,\alpha _3,\alpha _4,\alpha _5,\alpha _6,\alpha _7,\alpha _8,\alpha _9,\alpha _{10}) \approx (3.85,2.29,10.37,0.10,2.7,8.99,3.00,10.42,1.81 \times 10^{-6}, 0.50, 0.03)\\ (\beta _0,\beta _1,\beta _2,\beta _3) \approx (19.77,30.24,8.77,9.15) \end{array} \right. \end{aligned}$$

#### Comparison of the generated and the measured trajectories

Trajectories between the same starting and goal positions than the average trajectories were generated using the OC model with the computed optimal weights. Two examples are shown in Fig. 8a. Then, the linear and angular distances between the generated and the average trajectories were computed. For all the forward paths and for both subjects, the average distances were $\bar{d}_{xy} = 0.26 \pm 0.11 \, \mathrm{m}$ and $\bar{d}_{\theta } = 0.51 \pm 0.6 \, \mathrm{rad}$. For all the return paths, they were $\bar{d}_{xy} = 0.44 \pm 0.36 \, \mathrm{m}$ and $\bar{d}_{\theta } = 0.93 \pm 1.17 \, \mathrm{rad}$. The boxplots showing the distances per subjects and per direction are represented in Fig. 8b. Those linear and angular distances showed significant differences (Kruskal-Wallis test, $p < 0.05$) with the linear and angular distance between the average and the measured trajectories during Scenario 3 for both subjects. Thus, the gap between the model and the reality was greater than the variability of the measurements. Moreover, as the final constraint was weak, the model can generate trajectories which did not exactly reach the goal position. It is the case for 3 different trajectories plotted in Fig. 8a. Thus, we computed the euclidean distances between the goal position and the final position of each generated trajectory. The distances in average were $0.19 \, \mathrm{m}$ and $0.59 \, \mathrm{rad}$ for the forward paths and $0.26 \, \mathrm{m}$ and $1.24 \, \mathrm{rad}$ for the return paths.

## Discussion

To summarize, in this paper, we performed experiments to record CoM trajectories of pairs carrying a table. During those experiments, the 20 pairs were asked to move a table to nine different goals according to three scenarios. Their forward and return paths were both recorded. Thus, more than 3000 CoM trajectories were measured as part of this study. Then, all the retrieved data were processed and analysed in order to understand if a shared strategy emerges from all those experiments. In this context, a metrics was built to assess the differences between the measured trajectories. Moreover, a coupled OC model was designed to generate human-like CoM trajectories of two human carrying a table together. The method applied was based on the one Maroger et al.^22^ used to design a universal trajectory model for single walking humans. Finally, the generated trajectories were compared to the measured trajectories to assess the accuracy of the coupled OC model. All the data and the codes to analyse and model the walking pairs are open-source and available on: https://github.com/imaroger/pair_during_collaborative_carriage.

First of all, let us focus on the main question the data analysis aimed to answer: Is there a shared strategy implemented by the pairs to move a table around? The first conclusion drawn from the data analysis was that, during Scenario 3, 60% of the subjects chose a trajectory closer to the one they performed when they were the only one to know the goal position. However, this result could not be generalized to all the pairs. Thus, leadership was not the only determinant of the strategy implemented by a pair. Indeed, other results showed that the choices of configuration (i.e. the final orientation of the table for a given goal position) made when both partners were leaders were not systematically the same as the one made when Subject 1 was leader or when Subject 2 was leader. This leads to the conclusion that, broadly speaking, the strategy chosen by a pair did not depend on who knew where the table should be placed. The leadership only impacted the travel time, if both partners knew the goal position the task was sped up.

Then, it can be stated that the subjects did not choose the same trajectories when they carried a table from one start to one goal comparing to when they carried it backwards. Thus, the pairs’ strategy changed according to who walked forwards and who walked backwards.

Another conclusion resulting from the data analysis was that the pairs chose the optimal configuration for the whole pair in more than 80% of the experiments even if it was, most of the time, at the expense of the same member of the pair. Besides, it is still interesting to denote that the privileged subject was Subject 1, the one who walked forwards. This may be due to the fact that this subject was in average further from the goal position (see Fig.1a) and so the optimal strategy involved reducing its travelled distance. This means that a shared strategy aiming to reduce the travelled distance of the whole pair globally emerged from the experiments. However, it still means that, in around 20% of the experiments, pairs did not follow this strategy. The experimental conditions did not allow to understand the reason why some pair acted in a sub-optimal manner for their whole group while most did. So this strategy was implemented by the majority of the pairs, but it was not universal.

Finally, the comparison between the average trajectories and the measured trajectory shows that a great range of behaviour existed. As the various trajectories shown in Fig. 3a,b could indicate, when walking with a table, the pairs took very different trajectories which made the average trajectory barely representative. Even when looking at one pair, significant differences can be noted between trajectories performed from the same start to the same end. All those results suggested that a shared optimal strategy which would be implemented by every pair to carry a table around did not seem to exist. However, it is important to outline that there was one same choice that every subject made: each member of the pair chose to face the table instead of standing its back to it. Except from this choice, all the choices made by the pairs did not seem to follow a shared strategy. As the trajectories of single walking humans already presented a great variability^22^, it is not surprising that with two coupled humans the observed variability was even bigger. Indeed, the linear distances between the trajectory measured as part of this study and their respective average were about two times higher than the distances between the trajectories of single walking humans and their average ($0.11 \pm 0.06 \, \mathrm{m}$ for the linear distance and $0.48 \pm 0.18 \, \mathrm{rad}$ for the angular distance)^22^. Thus, we can conclude that the variability of human trajectories increased when two humans were interacting with each other. This is consistent with the results of some studies about collaborative work which demonstrated that individuals were less efficient when performing a task as a group than when performing alone^30,31^. Moreover it is interesting to denote that the variability of the table trajectories was lower than the variability of the subjects trajectories.

Thus, in this context we can state that no universal strategies emerged for carrying a table. Indeed, even if some shared strategies emerged from the experiments, none was implemented by every pairs. Moreover, the large trajectory profiles and the different configurations observed tended to demonstrate that there was not one optimal path followed by every pairs. In other words, if such optimal trajectories existed, the subjects were not able to find and follow them. Pairs did not demonstrate an optimal behaviour. Let us denote, that the result may be different if the experiments were performed by trained participants such as professional movers who know their partner well.

This great variability may explain the hardship to get accurate results when modeling the CoM trajectories of two humans walking with a table. In Maroger et al.^22^, an IOC scheme was used to build an OC model which accurately fits average CoM trajectories of single walking humans. In this paper, we applied the exact same method to investigate if an identical approach could provide similar results and build a coupled OC model to simultaneously simulate two humans handling a table. The generated trajectories could be considered as consistent as they are, most of the time, included in the corridors of observed trajectories. However, this model could not succeed in generating accurate human-like CoM trajectories. Indeed, we hypothesized that the model can be considered as accurate if the mean distances between the generated and the average measured trajectories are of the same order of magnitude than the mean distances between the individual and the average measured trajectories. According to this assumption, even if the results for the forward path along the $\vec {x}$ and $\vec {y}$ axis were suitable, the results for orientations and the return paths were not accurate enough. Thus, the starting hypothesis stating that we could model pairs of humans carrying a table with the same method used to model single walking humans turned out to be wrong. Indeed, the method, which worked well to model^22^ and even predict^23^ a single human behaviour, did not succeed in building an accurate model for two humans walking with a table. It was not totally unexpected as two humans carrying a table were not two independent holonomous systems coupled through a table. Their interactions may not just be haptic interactions but some other non-verbal interactions may play a role during the carriage task. For example, they may induce sudden changes in the implemented strategy resulting in unusual behaviours. As it could be expected, those potential interactions were not taken into account in the OC model. Moreover, the inaccurate results may also be due to the great range of behaviour observed during the experiments. The different average trajectories may reflect inconsistent behaviours which resulted in an impossible fitting of the average trajectories with on single set of weights. However, when trying to fit the individual measured trajectories performed by one pair instead of the average trajectories, this problem remained as one pair also displayed a too great range of behaviours as Fig.7c shows. Moreover, as better results were obtained for the forward path, the hypothesis made for the return paths may be wrong: the subjects’ roles may not be switched between the forward and the return paths. One solution may be to compute time-varying weights in order to identify changes in the subjects’ behaviour. Changing the dynamics of the system to model both subjects as a mass spring system might also be better than coupling the subjects with weak constraints put in the cost function. Indeed, low weights were assigned to the exponential barriers implemented in this manuscript. This means that the constraints they represented were not always observed. Another solution might be to remove outlier trajectories before computing the average trajectory. This might provide a model which fits the trajectories of pairs which have the most homogeneous behaviour. Thus, the main conclusion of this inaccurate modeling was that two interacting individuals cannot be modelled using the same optimization criteria that were used to model a single individual. The haptic interactions seemed to induce a change in the subjects’ behaviour which made them fundamentally different from one single walking subject and, thus, less predictable. To conclude, during the recorded interaction, the collaborating subjects set aside their individual behaviour to achieve a less predictable collective behaviour. As the model was unable to generate accurate trajectories for at least one scenario, we chose not to present the results regarding Scenario 1 and 2 as we can already state that this approach was not relevant to model pairs during collaborative carriage tasks.

In future works, the focus will be on modeling only the trajectory of the table using the same method as in Maroger et al.^22^. This must be more successful than trying to model the human trajectories that are too varied. Indeed, the data analysis showed a lesser variability regarding the table trajectories. Thus, the solution to accurately model the behaviour of a human during a table handling task may be able to model the table behaviour first and then to infer the carriers’ behaviour. Then, a prediction model may be developed based on this trajectory model as it was done in Maroger et al.^23^ to predict in real-time single walking human trajectories. However, it is important to denote that other approaches, which do not assumed the existence of an optimal trajectory, could have been considered to analyze the performed experiments. For example, the uncontrolled manifold analysis^32^, or other dynamical approaches^33^, could have also been applied here, perhaps, using the position of the table or its orientation as the performance variable.

Finally, a few remarks needed to be made on the experiments. A total of 3240 CoM trajectories were performed as part of this study. However, due to markers loss, 1.7% of the data were unprocessed. For every subject, 100% of the forward paths and 96.7% return paths were collected. Moreover, as mentioned in the data collection section, markers were put on the feet and on the head of the subjects. Thus, the database created from the performed experiments includes data that have not been analyzed yet. So, future works could study those remaining data that might provide new insights on the results especially regarding the step length or the orientation of the gaze during the carriage task. Furthermore, the experimental protocol could be improved. Indeed, as pairs always performed Scenario 1 before Scenario 2, some results cannot be easily interpreted. For example, the experiments during Scenario 2 were performed a little faster than the ones during Scenario 1. It is hard to say if this slight difference may be due to the change of leadership or just to the fact that the scenarios were performed one after the other. In other words, it remains unclear if a learning process emerged between the scenarios. It would be relevant to make new experiments where the different scenarios will be carried out in a random order. However, as the variability observed during the different scenarios is not significantly different and 30.5% of the pairs did not put the table in the same configuration during the 3 scenarios, we assumed that no learning process occurred between the different trials. Thus, the non-randomization of the scenarios is a clear limitation of the experimental protocol but it may have only a minor impact on the results of this study. Another improvement could be added to the experimental protocol. In these experiments, the subjects were asked to stay silent in order to avoid non-haptic collaborations. However, even if verbal communication was forbidden, no action was taken to avoid non-verbal communication like the gazes. To remove this bias in a future experiments, subjects should not see each other eyes, they should wear masks or be separated by a board placed in the middle of the table.

## Conclusion

To conclude, this manuscript provided a study of the trajectories performed during table handling tasks. As part of this work, 20 pairs of subjects performed 54 forward and return paths between various starting and goal positions according to different scenarios. Thus, more than 3000 CoM trajectories of the subjects and of the table were recorded and analysed. This analysis demonstrated the great variability in the choices made by the pairs to move a table around. No shared strategy, that all pairs would implement, emerged from those experiments. The only choice made by every subject was to face the table during the task instead of turning their back to it. Regarding the chosen trajectories or configurations, a great range of behaviours was observed. Because of this variability, simultaneously generating the CoM trajectories of the two members of a pair carrying a table was much more complex than generating the CoM trajectories of a single walking human. Thus, even if the simulated paths are, most of the time, included in the corridors of observed trajectories, the OC model introduced and optimized with an IOC scheme in this paper did not succeed in accurately simulating the pairs locomotion. Future work will focus on improving this model.

## Data Availability

The datasets generated and analyzed during the current study are available in the following Github repository: https://github.com/imaroger/pair_during_collaborative_carriage.
